# Predictors of illness course and health maintenance following inpatient treatment among patients with anorexia nervosa

**DOI:** 10.1186/s40337-020-00348-7

**Published:** 2020-12-02

**Authors:** Deborah R. Glasofer, Alexandra F. Muratore, Evelyn Attia, Peng Wu, Yuanjia Wang, Hillary Minkoff, Teresa Rufin, B. Timothy Walsh, Joanna E. Steinglass

**Affiliations:** 1 Department of Psychiatry, Columbia University Irving Medical Center, New York State Psychiatric Institute, 1051 Riverside Drive, Unit 98, New York City, NY 10032 USA; 2 Center for Eating Disorders, Weill Cornell Medical College, 21 Bloomingdale Road, White Plains, NY 10605 USA; 3 Department of Biostatistics, Mailman School of Public Health, 722 West 168th Street, New York, NY 10032 USA; 4 Harvard T.H. Chan School of Public Health, 677 Huntington Avenue, Boston, MA 02115 USA

**Keywords:** Anorexia nervosa, Longitudinal study, Inpatients, Outcome, Body mass index, Duration of illness, Time course, Prognosis

## Abstract

**Background:**

Anorexia nervosa (AN) is a life-threatening psychiatric disorder associated with significant medical and psychosocial impairment. Hospital-based behavioral treatment is an effective intervention in the short-term. However, relapse rates following discharge are high and thus, there is a need to identify predictors of longitudinal outcome. The current study provides information regarding illness course and health maintenance among patients with AN over 5 years following discharge from an eating disorder inpatient unit.

**Methods:**

Participants were individuals with AN who were discharged from a specialized, inpatient behaviorally-based unit. Prior to discharge, height and weight were measured and participants completed self-report measures of eating disorder severity and general psychopathology (depression, anxiety, harm avoidance). Participants were contacted annually for self-report measures of weight, eating disorder severity and clinical impairment. Outcome was defined by illness course (body mass index (BMI) and clinical impairment during the 5 years) and health maintenance (categories of weight and eating disorder symptom severity) across follow-up, using all available data. Linear mixed models were used to examine whether demographic and clinical parameters at discharge predicted BMI and clinical impairment over time. Additional analyses examined whether these variables significantly influenced an individual’s likelihood of maintaining inpatient treatment gains.

**Results:**

One-hundred and sixty-eight individuals contributed data. Higher trait anxiety at discharge was associated with a lower BMI during follow-up (*p* = 0.012). There was a significant interaction between duration of illness and time, whereby duration of illness was associated with a faster rate of weight loss (*p* = 0.003) during follow-up. As duration of illness increased, there was a greater increase in self-reported clinical impairment (*p* = 0.011). Increased eating disorder severity at discharge was also associated with greater clinical impairment at follow-up (*p* = 0.004). Higher BMI at discharge was significantly associated with maintaining healthy weight across a priori BMI-based definitions of health maintenance.

**Conclusions:**

Weight status (higher BMI) and duration of illness are key factors in the prognosis of AN. Higher weight targets in intensive treatments may be of value in improving outcomes.

**Supplementary Information:**

The online version contains supplementary material available at 10.1186/s40337-020-00348-7.

## Plain English summary

Anorexia nervosa (AN) is a life-threatening psychiatric disorder. Hospital-based behavioral treatment is an effective intervention in the short-term, but relapse rates following discharge are high. Here, we provide information on clinical features at the time of leaving the hospital that are related to illness course and health maintenance over the following five years. Prior to discharge, height and weight were measured and patients completed questionnaires about their eating disorder and associated symptoms. Participants were then contacted annually to answer questions about their eating disorder and health. One-hundred and sixty-eight individuals participated in at least one annual assessment. Longer duration of illness predicted faster weight loss after discharge, and was related to greater self-reported day-to-day impairment over time. Higher weight at discharge was associated with maintaining healthy weight over the follow-up period. High anxiety at discharge was associated with lower BMI over the 5-years of follow-up, while increased eating disorder severity was associated with greater day-to-day impairment. Results of this longitudinal study highlight that weight status at discharge and duration of illness are key factors in the course of AN. While duration of illness is not modifiable at the time of presentation for care, discharge weight is. Higher weight targets in intensive treatments may be of value in improving long-term outcomes.

## Background

Anorexia nervosa (AN) is a serious psychiatric disorder associated with medical complications [1], impairments in psychosocial functioning [2, 3], and significant morbidity and mortality [4–7]. Behavioral treatment in intensive settings is effective in restoring weight and improving eating behavior and psychological functioning in the short-term [8, 9]. Recovery rates in AN differ depending on how recovery is defined, and range widely [10]. The rates of relapse following inpatient care are consistently high, with estimates ranging between 35 and 57% [10–13]. As AN is a not a common illness and long-term studies are few, more data are needed to fully understand the clinical factors that determine course of illness and promote health maintenance over time.

Several longitudinal studies have examined the course of illness in AN using different methodology and clinical parameters. Most [13–19], but not all [20], have identified longer duration of illness as a predictor of worse long-term outcome. Body mass index (BMI) is also related to outcome: most studies show associations between lower admission and discharge BMI and worse outcomes in adults [11, 14, 16, 17, 21–25], though research in adolescents suggests discharge BMI is less predictive of outcome when inpatient treatment is followed by structured outpatient treatment [26, 27]. AN subtype has also been associated with outcome, though this has not been a consistent finding [18, 28]. Compared to those with restricting subtype, some research suggests that patients with the binge-purge subtype appear more likely to drop out of treatment prematurely and may be at higher risk for relapse [12, 19, 29–32].

The impact of psychological factors such as depression, anxiety, harm avoidance, eating disorder psychopathology, and motivation for recovery have been mixed [12, 13, 33–38]. Though multiple longitudinal studies have examined the course of AN, the majority of studies focus on predictors and correlates of outcomes such as diagnostic status, mortality, and rates of relapse [5, 7, 10–12, 15, 16, 31, 33–36, 39–41]. Prior studies on inpatient samples specifically have tended toward examining predictors of outcome unrelated to treatment (e.g., age of onset, comorbidities, family support) and report overall prognosis categorically [42–47]. Fewer have studied continuous outcomes, such as weight, symptoms, and overall clinical impairment over time [13, 23, 38, 48, 49], and these have generally been limited to short-term follow up [13, 19, 49] or to a single assessment point [23, 25, 34]. Together, these studies suggest that higher BMI at discharge and shorter duration of illness are important factors in the course of AN. Evaluation of trajectory of change and other clinical measures across multiple time points is needed to better understand variables that predict both the course of illness and prognosis following behaviorally-based inpatient treatment.

The present study examined the association between clinical features at the time of hospital discharge and longer-term outcome among adolescents and adults with AN, the majority of whom had achieved weight restoration to a BMI within the normal range (18.5–24.9 kg/m^2^). A longitudinal design, which allows for the examination of within-subject weight changes over multiple follow-up periods, was used to examine illness course over 5 years, and maintenance of health. We hypothesized that duration of illness and lower BMI at the time of hospital discharge would predict poorer longer-term outcome, and explored whether other clinical and psychological features impact outcome.

## Methods

All patients with AN discharged from inpatient treatment at the New York State Psychiatric Institute (NYSPI) Eating Disorders Research Unit (EDRU) are offered enrollment in this ongoing 10-year longitudinal study. Participants are contacted annually following hospital discharge. The current study is an examination of data collected over the first 5 years following discharge, using all available data from patients enrolled beginning in 2009. This study was reviewed and approved by the NYSPI Institutional Review Board. All participants provided written informed consent (individuals under age 18 provided assent and a parent or guardian provided consent).

### Participants

Participants were patients with AN who had been discharged from the EDRU between September 2009 and June 2018 and provided follow-up information on at least one occasion over the subsequent 5 years. Of the 196 patients discharged during this timeframe, 194 (99%) consented to participate. Diagnosis was established at hospital admission using the *Eating Disorder Assessment for DSM-5* (EDA-5) [50] or *Structured Clinical Interview for DSM-IV Axis I Disorders* (SCID-IV) [51], which also assessed co-occurring psychiatric illness.

### Procedures

Treatment at the EDRU is behaviorally-based and aims for full weight restoration [52], corresponding to a BMI of ~ 20.0 kg/m^2^ [9]. Prior to discharge, height and weight were measured and participants completed self-report measures of eating disorder severity (Eating Disorder Examination-Questionnaire – EDE-Q) [53], clinical impairment related to eating disorder symptoms (Clinical Impairment Assessment – CIA) [54], mood (Beck Depression Inventory – BDI) [55] and anxiety symptoms (Beck Anxiety Inventory – BAI) [56] and anxious traits (Spielberger State Trait Anxiety Inventory-Trait version – STAI-T) [57] and temperament (Temperament and Character Inventory – Harm Avoidance subscale – TCI-HA)) [58].

Procedures included annual phone interviews with participants and administration of self-report questionnaires. Weight was obtained by self-report during each annual assessment, in the context of an interview on psychosocial functioning, weight history (since last contact), current eating behavior, and medical status. In addition, patients were asked to provide permission for contact with a clinician to verify the reported weight. Clinician-reported weights were used when available; otherwise, self-reported weights were used. Self-report measures of eating disorder severity (EDE-Q) and eating disorder related psychosocial impairment (CIA) were sent for participant completion. Participants were paid $10 after completion of study questionnaires.

### Data analysis

Data from a follow-up assessment were excluded if the individual had been receiving more than one week of structured treatment for AN (e.g., inpatient treatment, residential treatment; *n* = 10), or met criteria for bulimia nervosa or binge-eating disorder (*n* = 8) at the time of the assessment (as by BMI alone these individuals might otherwise have been considered healthy). Independent samples t-tests and Pearson chi-square tests were used to compare participants and non-contributors on demographic and clinical characteristics. Self-reported weights and clinician weights were compared using Pearson correlations. Independent samples t-tests compared demographic and clinical characteristics between individuals for whom both self-reported and clinician-reported weights were available and individuals for whom there was only one weight.

#### Illness course

Primary analyses examined BMI and CIA score (outcomes) at each year of follow-up with a linear mixed effects model fitting all available data. Age, time (year of follow-up), subtype, sex, duration of illness, BAI, BDI, STAI-T, EDE-Q (Global score), and TCI-HA were included as covariates in both models. Interaction between duration of illness and time was included to assess whether duration of illness predicted rate of change in BMI or CIA over 5 years. The number of annual assessments completed by each individual was included in both models to adjust for different number of observations available for each individual. Additionally, CIA at discharge was included as a predictor of BMI, and BMI at discharge was included as a predictor of CIA. Discharge BMI and discharge CIA were included as baseline (Year 0) outcomes and were therefore not included as predictors of BMI or CIA in their respective models. Of the total sample, 101 and 104 individuals had complete predictor data at discharge for inclusion in BMI and CIA analyses, respectively.

#### Health maintenance

To specifically assess factors associated with maintaining health after hospitalization, we tested the ability of the same variables to predict health maintenance (“good outcome”) using a logistic regression model (TCI-HA was not included in this analysis due to small sample sizes in each category and missing data.). Health maintenance was defined in 3 ways: (1) maintenance of a BMI ≥ 18.5 kg/m^2^ at all available time points, (2) maintenance of a BMI ≥ 19.5 kg/m^2^ at all available time points, (3) maintenance of a BMI ≥ 18.5 kg/m^2^ and an EDE-Q score of ≤ 2 at all available time points (maintenance of a BMI ≥ 19.5 kg/m^2^ and an EDE-Q score of ≤ 2 at all available time points was not included due to insufficient data). BMI cutoffs were selected based on suggested diagnostic thresholds [59] and suggested recovery thresholds per previous reports [60, 61]. EDE-Q score cutoffs were selected to be within 1 SD of Global EDE-Q norms for young adult women [62], and consistent with existing conceptualizations of eating disorder health maintenance [60, 61]. Logistic regression models were fitted to the data to test whether there was a relationship between a patient’s score on each variable and the likelihood of meeting criteria for each of the three health maintenance groups at follow up. Of the patients meeting criteria for health maintenance, three had been discharged from the hospital before reaching a BMI of 18.5 kg/m^2^; as such, these individuals had *gained* weight, not *maintained* weight. Sensitivity analyses were conducted without these three participants.

SAS software, version 9.4, and SPSS, version 26.0, were used for all statistical analyses and modeling. Alpha level was set to 0.05.

## Results

### Demographics, clinical characteristics and response rate

One-hundred ninety-four patients with AN (176 adults, 18 adolescents) were enrolled in the study, of whom 24 (12.4%) did not participate in any post-hospitalization assessments. Of the remaining 168, 42 individuals reported information at one time point, 31 individuals reported information at two time points, 35 individuals reported information at three time points, 26 individuals provided information at four time points, and 34 individuals provided information at five time points. Data from these 168 individuals were used in analyses (Table 1). Of these individuals, twenty participants (11.9%) were discharged at a BMI < 18.5 kg/m^2^. Co-occurring diagnoses included current or past anxiety disorders (*n* = 62, 37%), major depressive disorder (*n* = 80, 48%), obsessive-compulsive disorder (*n* = 17, 10%), post-traumatic stress disorder (n = 17, 10%), and substance use disorders (*n* = 22, 13%) (Complete comorbidity data were missing for 11 participants).

**Table 1:** Demographic and Clinical Characteristics

	Participants ***N*** = 168	
**Demographic Variables**	**Mean**	**Range**
Age (years)	26.02 ± 8.20	14–64
Sex, female, *n* (%)	164 (98%)	n/a
Race, Caucasian, *n* (%)	148 (88%)	n/a
AN subtype, binge-purge, *n* (%)	82 (49%)	n/a
Duration of hospitalization (days)	78.82 ± 43.22	0–462
Duration of illness (years)	9.36 ± 7.70	0.25–50
Admission BMI (kg/m^2^)	16.00 ± 1.86	10.7–20.8
Discharge BMI (kg/m^2^)	20.02 ± 1.46	14.6–23.4
**Psychological Measures – Discharge**	**Mean** ± **SD**	**Range**
BAI (*n* = 135)	18.45 ± 11.45	0.00–61.00
BDI (*n* = 132)	17.89 ± 11.84	0.00–51.00
STAI-T (*n* = 131)	56.89 ± 12.18	25.00–80.00
CIA (*n* = 118)	23.53 ± 11.51	3.00–48.00
EDE-Q Global (*n* = 122)	2.67 ± 1.26	0.19–5.05
TCI-HA (*n* = 117)	21.60 ± 7.07	4.00–34.00

*BMI* body mass index. *BAI* Beck Anxiety Inventory, *BDI* Beck Depression Inventory, *STAI-T* Spielberger State Trait Anxiety Inventory-Trait version, *EDE-Q* Eating Disorder Examination – Questionnaire*. TCI-HA* Temperament and Character Inventory, Harm Avoidance Subscale

The group that did not provide any follow up data (i.e., non-contributors) did not differ significantly in age, sex, race, admission BMI, or duration of illness (see Supplemental Table 1). Compared with participants, the non-contributors had a significantly lower BMI at discharge and a greater proportion of individuals with AN binge-purge subtype. Among the 168 participants, 56.5–67.3% provided BMI data each year (see Table 2). Of 457 total weights provided, 147 included both self-reported and clinician-verified weights. When weights from both sources were available, self-reported weights were highly correlated with clinician-provided weights (*r* = .970, *p* = .000). Participants who had weights from both sources and those who had weights from one source did not differ in age, weight, duration of illness, duration of hospitalization, admission BMI or discharge BMI (*t*s ≤± 1.82, *p*s = ≥ .069).

**Table 2:** Number of Individuals Providing Data at each Annual Assessment

	Year 1	Year 2	Year 3	Year 4	Year 5
Total Number Participants Eligible to Provide Information at Time Point	168	160	147	129	112
Number Participants Who Provided BMI (*n*, %)	113 (67%)	101 (63%)	98 (67%)	73 (57%)	71 (63%)
Number Participants Who Provided CIA (*n*, %)	88 (52%)	75 (47%)	74 (50%)	62 (48%)	55 (49%)
Number Participants Who Provided EDE-Q Global (*n*, %)	89 (53%)	81 (51%)	78 (53%)	73 (57%)	58 (52%)

Table listing the proportion of individuals who provided data at each of the five follow-up time points (Year 1-Year 5). Row 2 lists the total number of individuals who had reached the time point for data collection, and were therefore eligible. Rows 2–4 show the proportion of participants at each time-point who provided information with regard to body mass index (BMI), Clinical Impairment Assessment (CIA), and Eating Disorder Examination – Questionnaire (EDE-Q), respectively. Proportion of individuals who provided information are listed as numbers (n) and percent of total who were eligible (%)

### Primary analyses: longitudinal illness course

Of the 168 participants, 101 and 104 individuals had complete predictor data for BMI and CIA analyses, respectively. Table 3 reports results of linear mixed effects model analyses with BMI and CIA as outcomes. There was a significant interaction between time and duration of illness, such that as Duration of Illness increased, there was a greater decrease in BMI over time – meaning, a steeper slope of weight loss following discharge (*F*(1,284) = 13.34, *p* = .0003). Higher STAI-T score at discharge was associated with lower BMI during the follow-up timeframe (*F*(1,284) =6.36, *p* = .012). Higher TCI-HA score at discharge was associated with higher BMI across time-points, *F*(1,284) = 3.97, *p* = .047. No other variables were associated with BMI. In the model predicting CIA, there was a significant interaction between time and duration of illness, such that as Duration of Illness increased, there was a greater increase in CIA scores (i.e., greater self-reported impairment) over time, *F*(1,232) = 6.65, *p* = .011. Time was associated with CIA, such that across follow-up there was an overall decrease in CIA scores, *F*(1,232) = 17.13, *p* < .0001. The EDE-Q Global score was associated with CIA across time-points, with higher EDE-Q scores at discharge predicting increased CIA scores during follow-up (*F*(1,232) = 8.38, *p* = .004).

**Table 3:** Fixed Effects from Linear Mixed Model of Longitudinal Outcome

Variables	BMI (*n* = 101)				CIA (*n* = 104)			
	*F*	*ß*	*SE ß*	*p*	*F*	*ß*	*SE ß*	*p*
Intercept	–	21.959	1.326	<.0001	–	15.749	18.216	0.389
Time	3.55	0.178	0.094	0.060	17.13	−2.179	0.527	**< 0.0001**
AN subtype, binge-purge	2.31	0.565	0.372	0.129	0.01	0.209	1.756	0.905
Sex, female	0.02	0.132	0.922	0.887	0.01	0.328	4.424	0.941
Duration of Illness (years)	1.48	−0.038	0.032	0.225	0.78	0.136	0.154	0.378
CIA at Discharge	0.28	0.015	0.029	0.598	–	–	–	–
Discharge BMI (kg/m^2^)	–	–	–	–	1.15	−0.851	0.793	0.284
BAI	2.59	0.034	0.021	0.109	1.56	0.124	0.099	0.213
BDI	0.33	−0.013	0.024	0.566	0.23	0.049	0.103	0.635
EDE-Q Global	0.15	−0.099	0.258	0.701	8.38	2.934	1.013	**0.004**
TCI-HA	3.97	0.061	0.030	**0.047**	0.01	−0.013	0.141	0.929
STAI-T	6.36	−0.071	0.028	**0.012**	2.68	0.213	0.129	0.103
Time x Duration of Illness	13.34	−0.033	0.009	**0.0003**	6.65	0.131	0.051	**0.011**

*BAI* Beck Anxiety Inventory, *BDI* Beck Depression Inventory*, BMI* body mass index, *CIA* Clinical Impairment Assessment, *EDE-Q* Eating Disorder Examination - Questionnaire, *STAI-T* Spielberger State Trait Anxiety Inventory-Trait version, *TCI-HA* Temperament and Character Inventory, Harm Avoidance Subscale. Bold values = *p* < 0.05

### Health maintenance

Of the 168 participants, 77 individuals (45.8%) maintained a BMI ≥ 18.5 kg/m^2^ at all available time points post-hospitalization, 54 maintained (32.1%) a BMI ≥ 19.5 kg/m^2^, and 29 (17.3%) maintained a BMI ≥ 18.5 kg/m^2^ and EDE-Q scores ≤ 2. After restricting participants to those with complete data at discharge (for model analyses), the sample included 53 (46.1%) with BMI ≥ 18.5 kg/m^2^, 37 (34.8%) with BMI ≥ 19.5 kg/m^2^, and 20 (13.2%) with BMI ≥ 18.5 kg/m^2^ and EDE-Q scores ≤ 2. Table 4 provides Odds Ratio (OR) and 95% Confidence Intervals for the relationship between predictor variables and likelihood of meeting criteria for each of the three categorical outcomes.

**Table 4:** Odds Ratios and Confidence Intervals for the Relationship between Predictors and Clinical Outcomes

	Outcome Category 1 BMI ≥ 18.5 (*n* = 53)			Outcome Category 2 BMI ≥ 19.5 (*n* = 37)			Outcome Category 3 BMI ≥ 18.5 and EDE-Q ≤ 2 (*n* = 20)		
	Odds Ratio	95% CI	*p*	Odds Ratio	95% CI	*p*	Odds Ratio	95% CI	*p*
Duration of Illness (years)	0.973	0.903–1.048	0.471	0.916	0.837–1.002	0.055	0.877	0.772–.996	**0.043**
BAI	1.026	0.973–1.082	0.345	1.041	0.976–1.111	0.225	1.005	0.922–1.095	0.914
BDI	0.956	0.900–1.015	0.139	0.927	0.854–1.005	0.066	0.953	0.854–1.063	0.385
STAI-T	1.009	0.950–1.072	0.769	1.019	0.953–1.090	0.586	1.043	0.950–1.144	0.377
EDE-Q Global	0.870	0.508–1.488	0.611	0.563	0.296–1.070	0.079	0.289	0.106–.788	**0.015**
AN subtype, binge-purge	1.148	0.425–3.100	0.785	0.857	0.268–2.736	0.794	0.319	0.074–1.380	0.126
Admission BMI (kg/m^2^)	1.680	1.215–2.323	**0.002**	1.294	0.911–1.838	0.149	1.256	0.812–1.943	0.307
Discharge BMI	2.211	1.189–4.112	**0.012**	3.180	1.589–6.365	**0.001**	1.923	.925–3.996	0.079
Number of Annual Assessments Completed	0.740	0.514–1.065	0.105	0.426	0.255–.711	**0.001**	0.715	0.411–1.246	0.237

*BAI* Beck Anxiety Inventory, *BDI* Beck Depression Inventory, *BMI* body mass index, *EDE-Q* Eating Disorder Examination – Questionnaire, *STAI-T* Spielberger State Trait Anxiety Inventory-Trait version. Bold values = *p* < 0.05. Reported ns refer to number of individuals who met criteria for each outcome category

Higher BMI at discharge significantly increased the likelihood of meeting criteria for two of the “good outcome” categories (Table 4). Specifically, a one unit increase in BMI at discharge led to a 2.21-fold increase in the odds of maintaining a BMI ≥ 18.5 kg/m^2^ and a 3.18-fold increase in the odds of maintaining of a BMI ≥ 19.5 kg/m^2^. Higher admission BMI also increased the likelihood of weight maintenance at follow-up, though the effect was less pronounced.

The number of annual follow-up assessments patients completed led to a 0.43-fold decrease in the odds of a participant maintaining a BMI of ≥ 19.5 kg/m^2^, but did not contribute significantly to meeting criteria for the other categories. A sensitivity analysis was conducted including only participants who completed ≤ 4 annual assessments, and the pattern of results did not change (0.49-fold decrease in the odds of maintaining a BMI of ≥ 19.5 kg/m^2^).

Longer duration of illness significantly decreased the likelihood of maintaining BMI of ≥ 18.5 kg/m^2^ and an EDE-Q Global Score of ≤ 2 across all time points. Specifically, for every year increase in duration of illness, there was a 0.88-fold decrease in the odds of a participant maintaining weight within a normal range and reduced eating disorder symptoms. Higher EDE-Q score at discharge significantly decreased the likelihood of meeting criteria for this category, such that a one-point increase in EDE-Q Global score led to a 0.29-fold decrease in the odds of a participant maintaining a BMI ≥ 18.5 kg/m^2^ and an EDE-Q score ≤ 2.

There were no differences in results of health maintenance analyses conducted with and without the three patients who did not achieve a BMI of 18.5 kg/m^2^ prior to discharge.

## Discussion

In this study, data collected post-hospitalization for 5 years from 168 patients with AN were examined to identify clinical factors that impact the course of illness. Two main factors emerged, one modifiable and one non-modifiable: BMI at discharge and duration of illness, respectively. More specifically, longer duration of illness was associated with more rapid weight loss after discharge and greater increase in reported clinical impairment, and higher BMI at discharge was associated with maintaining healthy weight. In addition, trait anxiety at discharge was associated with lower BMI across the 5 years of follow-up and surprisingly, harm avoidance at discharge was associated with higher BMI across the follow-up period. EDE-Q at discharge was associated with greater clinical impairment across the 5 years of follow-up.

The literature is reasonably consistent in finding that the BMI reached in treatment affects prognosis [13, 14, 21, 25, 63]. Although research with adolescents suggests lower discharge weight does not negatively influence outcomes [26, 27], individuals in these studies were discharged directly to a structured outpatient treatment, which likely influenced outcomes. This finding may also be specific to adolescents rather than adults, who comprise the majority of the current sample. As BMI is one of few potentially modifiable factors, the finding in the current study is important for treatment settings to consider. The majority of patients in this study (89.7%) were discharged from the hospital after full weight restoration. Excluding the twenty patients who left prior to achieving a BMI of ≥18.5 kg/m^2^, the range of BMI at discharge was 19.0 to 23.4 kg/m^2^, and in the health maintenance analyses, even including only patients in this normal range, a higher BMI at discharge predicted maintenance of a good outcome by two of the three definitions used. This is consistent with a prior study of weight-restored patients with AN (BMI = 19.2–22.0 kg/m^2^, mean = 20.3 kg/m^2^), which found that a higher BMI at the time of entry into relapse prevention treatment was associated with successful weight maintenance [13]. In an analysis of archival data of adults with AN participating in several outpatient treatment trials, weight restoration to above 85.8% of ideal body weight by the end of treatment was the best predictor of recovery (defined as a BMI > 19 kg/m^2^, eating disorder severity scores within 1 standard deviation of community norms, and the absence of binge-purge behaviors) at one year follow-up [64]. Prior results and current findings highlight the value of adequate weight restoration in promoting health among patients with AN.

In this study, adequate weight restoration was possible within the scope of our behaviorally-based inpatient program for research participants [52], where length of stay was not determined by insurance coverage or financial status. The significance of length of stay and its relationship with other predictors included in the current analyses may be substantially different when subject to the constraints of financial coverage. Given that the majority of treatment settings are subject to these constraints, and that length of stay has been shown to be impacted by service setting [65], our findings may be limited in generalizability. However, the potent impact of discharge BMI on outcome in our sample emphasizes the importance of identifying factors that predict which individuals will remain in care to complete weight restoration. The interventions most helpful in facilitating this treatment engagement will be as to improving long-term outcome.

This study replicates and extends existing literature suggesting that longer duration of illness is associated with worse outcome in AN [13–19]. Our findings highlight the relationship between a longer duration of illness and an increase in impairment related to the eating disorder over time, as well as an association with rate of weight loss after hospital discharge. The significant impact of duration of illness on prognosis presents a clinical conundrum: intensive treatment seems indicated, yet it is burdensome to provide if relapse rates are high. As duration of illness is not modifiable at the time of presentation for care, this factor may be more relevant for consideration of treatment algorithms, including decision-making about sequencing levels of care or other interventions. Some studies have identified the period following hospitalization as an especially high-risk period [13], and it may be that for individuals with a longer illness it will be particularly valuable to focus resources on this phase. The efficacy of “step down” care in preventing relapse may merit further research. The more favorable prognosis associated with shorter duration of illness [14, 18, 20, 34, 35, 39, 41, 66] suggests that early intervention may be important, as well.

The role of psychological variables in the course of AN is less consistent across studies [12, 13, 33–38]. In the current study, greater trait anxiety as measured by STAI, but not anxiety at discharge as measured by BAI, predicted lower mean BMI during the following 5 years. The measure of clinical impairment used in this study, CIA, specifically addresses eating-disorder related psychosocial impairment, and therefore not surprisingly was related to EDE-Q scores at discharge (both measures assess the frequency and interference of eating habits, exercise, and overvaluation of shape and weight [53, 54]). Yet, EDE-Q scores were not related to health maintenance. Psychological symptom severity may be less reliable in determining long term prognosis. The anxiety finding in this study is of some interest because there are data to suggest that anxiety precedes illness onset [67, 68], is related to illness severity [34, 69], and may be a useful treatment target in its own right [70, 71]. On the other hand, harm avoidance is also a measure of anxiety, and this was associated with higher BMI during follow-up. This discrepancy underscores that psychological measures may be less reliable prognostic markers. Contrary to previous research [12, 19, 29–32], we found no effect of AN subtype on illness course or outcome. However, the non-contributors had a significantly greater proportion of patients with binge-purge subtype, suggesting individuals with this subtype were less likely to provide follow-up data. It is therefore impossible to assess whether this discrepancy may be indicative of a systematic difference (i.e., differential outcome or rates of relapse) between subtypes not captured in the participating sample.

One challenge for the current study was setting the a priori definition of “good outcome.” A BMI of 18.5 kg/m^2^ is the suggested lower threshold of normal BMI in the general population, and is commonly used as a guide for “underweight” status in meeting criteria for AN [59]. There is some consensus within the eating disorders field that achieving BMI ≥ 18.5 kg/m^2^ is a required component of remission [72], and several studies support that this threshold is associated with sustained recovery [25, 73, 74]. In our sample, the rate of maintenance at or above a BMI of 18.5 kg/m^2^ for the duration of follow-up, from one to five years, was 46.1%, similar to or slightly higher than rates reported in previous studies [12, 24, 41]. Notably, only 17.4% of individuals maintained a BMI ≥ 18.5 kg/m^2^ and an EDE-Q score of ≤ 2. Clinically, patients with AN are commonly encouraged to consider a higher weight range in order to better mitigate other symptoms, and our data support this approach in that higher BMI reduced the risk of relapse. In the current study, the number of individuals who achieved a higher BMI in combination with lower EDE-Q scores was low, and so this study may not have been sufficiently powered to fully examine factors associated with this definition of health [60].

This study has several strengths. The vast majority of patients with AN admitted to the inpatient program agreed to participate and between one-half to two-thirds of those eligible responded to each annual follow-up. These annual assessments provide a rich longitudinal dataset of anthropometric and clinical measures during and in the years following inpatient weight restoration treatment for AN. The two statistical approaches are complementary in examining factors associated with illness course and with health maintenance.

The primary limitation of this study was the extent of missing data. Non-contributors in our sample were disproportionately individuals with binge-purge subtype who did not fully restore weight in the program, which could have introduced bias into the data. As the majority of individuals included in analyses had restricting subtype, it is possible our analyses do not fully capture the influence of binge-purge subtype on weight and clinical outcomes. Subtype was the sole characteristic that distinguished non-contributors from contributors. This is consistent with previous literature suggesting patients with binge-purge subtype may be more likely to drop out of treatment [29, 30] and may provide additional evidence suggesting a distinction between the two subtypes and their respective treatment outcomes. The assessments used in this study may have missed some potentially important variables with regard to long-term health, such as dietary intake patterns [75, 76], and it may be useful to obtain other measures of wellness, such as life satisfaction or health care utilization, to more broadly capture outcome. Though this study utilized clinician-reported weights where available, the majority of weights used in analyses were self-reported; this limitation is mitigated by the high correlation between clinician-reported and self-reported weights. Another notable limitation of this study is the finding that the number of annual assessments completed was associated with decreased likelihood of maintaining a weight of 19.5 kg/m^2^, suggesting some of our a priori definitions of “good outcomes” may be confounded by number of assessments completed. Future studies may wish to define “outcome” differently or using a fixed number of assessments to control for this bias. Finally, the primary aim of the current study was to examine how clinical status at discharge predicts illness course over time. As such, the present analyses did not examine how *changes* to these predictors over time influence outcome. Future research utilizing longitudinal datasets may wish to examine how changes to these predictors impact illness course.

## Conclusions

Longitudinal research conducted over an extended time period is critical to better understand illness course and prognosis in AN. Our findings reinforce the value of weight gain well within the normal range as a goal of treatment, and the importance of considering the role of duration of illness in overall prognosis and treatment planning.

## Supplementary Information

**Additional file 1:** **Supplemental Table 1.** Demographics and Clinical Characteristics of Participants and Non-Contributors

## Data Availability

The data supporting the results reported in this article are maintained by the Eating Disorders Research Unit of the New York State Psychiatric Institute. Please contact the corresponding author with data requests.

## Abbreviations

AN: Anorexia nervosa
BMI: Body mass index
NYSPI: New York State Psychiatric Institute
EDRU: Eating Disorders Research Unit
EDA-5: Eating Disorder Assessment for DSM-5
SCID-IV: Structured Clinical Interview for DSM-IV Axis I Disorders
EDE-Q: Eating Disorder Examination – Questionnaire
BDI: Beck Depression Inventory
BAI: Beck Anxiety Inventory
STAI-T: Spielberger State Trait Anxiety Inventory, Trait version
TCI-HA: Temperament and Character Inventory – Harm Avoidance Subscale
CIA: Clinical Impairment Assessment
OR: Odds Ratio

## References

[1] Mehler PS, Brown C (2015). Anorexia nervosa - medical complications. J Eat Disord.

[2] Mond JM, Owen C, Hay PJ, Rodgers B, Beumont PJV (2005). Assessing quality of life in eating disorder patients. Qual Life Res.

[3] Bohn K, Doll HA, Cooper Z, O'Connor M, Palmer RL, Fairburn CG (2008). The measurement of impairment due to eating disorder psychopathology. Behav Res Ther.

[4] Arcelus J, Mitchell AJ, Wales J, Nielsen S (2011). Mortality rates in patients with anorexia nervosa and other eating disorders. A meta-analysis of 36 studies. Arch Gen Psychiatry.

[5] Fichter MM, Quadflieg N (2016). Mortality in eating disorders - results of a large prospective clinical longitudinal study. Int J Eating Disord.

[6] Smink FR, van Hoeken D, Hoek HW (2013). Epidemiology, course, and outcome of eating disorders. Curr Opin Psychiatr.

[7] Franko DL, Keshaviah A, Eddy KT, Krishna M, Davis MC, Keel PK (2013). A longitudinal investigation of mortality in anorexia nervosa and bulimia nervosa. Am J Psychiatry.

[8] Olmsted M, McFarlane T, Carter J, Trottier K, Woodside D, Dimitropoulos G. The Treatment of Eating Disorders: A Clinical Handbook. 2010.

[9] Attia E, Walsh BT (2009). Behavioral Management for Anorexia Nervosa. N Engl J Med.

[10] Khalsa SS, Portnoff LC, McCurdy-McKinnon D, Feusner JD (2017). What happens after treatment? A systematic review of relapse, remission, and recovery in anorexia nervosa. J Eat Disord.

[11] Carter JC, Mercer-Lynn KB, Norwood SJ, Bewell-Weiss CV, Crosby RD, Woodside DB (2012). A prospective study of predictors of relapse in anorexia nervosa: implications for relapse prevention. Psychiatry Res.

[12] Carter JC, Blackmore E, Sutandar-Pinnock K, Woodside DB (2004). Relapse in anorexia nervosa: a survival analysis. Psychol Med.

[13] Kaplan AS, Walsh BT, Olmsted M, Attia E, Carter JC, Devlin MJ (2009). The slippery slope: prediction of successful weight maintenance in anorexia nervosa. Psychol Med.

[14] Errichiello L, Iodice D, Bruzzese D, Gherghi M, Senatore I (2016). Prognostic factors and outcome in anorexia nervosa: a follow-up study. Eating weight Disord.

[15] Lo Sauro C, Castellini G, Lelli L, Faravelli C, Ricca V (2013). Psychopathological and clinical features of remitted anorexia nervosa patients: a six-year follow-up study. Eur Eating Disord Rev.

[16] Vall E, Wade TD (2015). Predictors of treatment outcome in individuals with eating disorders: a systematic review and meta-analysis. Int J Eat Disord.

[17] Zipfel S, Löwe B, Reas DL, Deter H-C, Herzog W (2000). Long-term prognosis in anorexia nervosa: lessons from a 21-year follow-up study. Lancet.

[18] Wild B, Friederich H-C, Zipfel S, Resmark G, Giel K, Teufel M (2016). Predictors of outcomes in outpatients with anorexia nervosa – results from the ANTOP study. Psychiatry Res.

[19] Le Grange D, Fitzsimmons-Craft EE, Crosby RD, Hay P, Lacey H, Bamford B (2014). Predictors and moderators of outcome for severe and enduring anorexia nervosa. Behav Res Ther.

[20] Fichter MM, Quadflieg N, Crosby RD, Koch S (2017). Long-term outcome of anorexia nervosa: results from a large clinical longitudinal study. Int J Eat Disord.

[21] Wales J, Brewin N, Cashmore R, Haycraft E, Baggott J, Cooper A (2016). Predictors of positive treatment outcome in people with anorexia nervosa treated in a specialized inpatient unit: the role of early response to treatment. Eur Eat Disord Rev.

[22] Støving RK, Andries A, Brixen K, Bilenberg N, Hørder K (2011). Gender differences in outcome of eating disorders: a retrospective cohort study. Psychiatry Res.

[23] Strobel C, Quadflieg N, Naab S, Voderholzer U, Fichter MM (2019). Long-term outcomes in treated males with anorexia nervosa and bulimia nervosa—a prospective, gender-matched study. Int J Eat Disord.

[24] Casper RC, Jabine LN (1996). An eight-year follow-up: outcome from adolescent compared to adult onset anorexia nervosa. J Youth Adolesc.

[25] El Ghoch M, Calugi S, Chignola E, Bazzani PV, Dalle GR (2016). Body mass index, body fat and risk factor of relapse in anorexia nervosa. Eur J Clin Nutr.

[26] Madden S, Miskovic-Wheatley J, Wallis A, Kohn M, Lock J, Le Grange D (2015). A randomized controlled trial of in-patient treatment for anorexia nervosa in medically unstable adolescents. Psychol Med.

[27] Herpertz-Dahlmann B, Schwarte R, Krei M, Egberts K, Warnke A, Wewetzer C (2014). Day-patient treatment after short inpatient care versus continued inpatient treatment in adolescents with anorexia nervosa (ANDI): a multicentre, randomised, open-label, non-inferiority trial. Lancet (London, England).

[28] Zeeck A, Hartmann A, Buchholz C, Herzog T. Drop outs from in-patient treatment of anorexia nervosa. Acta Psychiatr Scand 2005;111(1):29–37. 10.1111/j.1600-0447.2004.00378.x 15636591

[29] Gregertsen EC, Mandy W, Kanakam N, Armstrong S, Serpell L (2019). Pre-treatment patient characteristics as predictors of drop-out and treatment outcome in individual and family therapy for adolescents and adults with anorexia nervosa: a systematic review and meta-analysis. Psychiatry Res.

[30] Kahn C, Pike KM (2001). In search of predictors of dropout from inpatient treatment for anorexia nervosa. Int J Eat Disord.

[31] Zipfel S, Lowe B, Reas DL, Deter HC, Herzog W (2000). Long-term prognosis in anorexia nervosa: lessons from a 21-year follow-up study. Lancet (London, England).

[32] Surgenor LJ, Maguire S, Beumont PJV (2004). Drop-out from inpatient treatment for anorexia nervosa: can risk factors be identified at point of admission?. Eur Eat Disord Rev.

[33] Franko DL, Tabri N, Keshaviah A, Murray HB, Herzog DB, Thomas JJ (2018). Predictors of long-term recovery in anorexia nervosa and bulimia nervosa: data from a 22-year longitudinal study. J Psychiatr Res.

[34] Zerwas S, Lund BC, Von Holle A, Thornton LM, Berrettini WH, Brandt H (2013). Factors associated with recovery from anorexia nervosa. J Psychiatr Res.

[35] Eddy KT, Tabri N, Thomas JJ, Murray HB, Keshaviah A, Hastings E (2017). Recovery from anorexia nervosa and bulimia nervosa at 22-year follow-up. J Clin Psychiatr.

[36] Keel PK, Dorer DJ, Franko DL, Jackson SC, Herzog DB (2005). Postremission predictors of relapse in women with eating disorders. Am J Psychiatry.

[37] Bulik CM, Sullivan PF, Fear JL, Pickering A (2000). Outcome of anorexia nervosa: eating attitudes, personality, and parental bonding. Int J Eating Disord.

[38] Segura-García C, Chiodo D, Sinopoli F, De Fazio P (2013). Temperamental factors predict long-term modifications of eating disorders after treatment. BMC Psychiatr.

[39] Andrés-Pepiñá S, Plana MT, Flamarique I, Romero S, Borras R, Julia L (2020). Long-term outcome and psychiatric comorbidity of adolescent-onset anorexia nervosa. Clin Child Psychol Psychiatr.

[40] Dobrescu SR, Dinkler L, Gillberg C, Rastam M, Gillberg C, Wentz E. Anorexia nervosa: 30-year outcome. Br J Psychiatr. 2019:1–8. 10.1192/bjp.2019.113 PMC7557598 31113504

[41] Fichter MM, Quadflieg N, Hedlund S (2006). Twelve-year course and outcome predictors of anorexia nervosa. Int J Eating Disord.

[42] Seidensticker JF, Tzagournis M (1968). Anorexia nervosa—clinical features and long term follow-up. J Chronic Dis.

[43] Morgan HG, Russell GF (1975). Value of family background and clinical features as predictors of long-term outcome in anorexia nervosa: four-year follow-up study of 41 patients. Psychol Med.

[44] Nussbaum M, Shenker IR, Baird D, Saravay S (1985). Follow-up investigation in patients with anorexia nervosa. J Pediatr.

[45] Bryant-Waugh R, Knibbs J, Fosson A, Kaminski Z, Lask B (1988). Long term follow up of patients with early onset anorexia nervosa. Arch Dis Child.

[46] Steinhausen H-C, Seidel R (1993). Outcome in adolescent eating disorders. Int J Eat Disord.

[47] Halmi K, Brodland G, Loney J (1973). Prognosis in anorexia nervosa. Ann Intern Med.

[48] Boehm I, Finke B, Tam FI, Fittig E, Scholz M, Gantchev K (2016). Effects of perceptual body image distortion and early weight gain on long-term outcome of adolescent anorexia nervosa. Eur Child Adolesc Psychiatr.

[49] Uniacke B, Attia E, Kaplan A, Walsh BT. Weight suppression and weight maintenance following treatment of anorexia nervosa. International journal of eating disorders. 2020. 10.1002/eat.23269 PMC7584398 32227368

[50] Sysko R, Glasofer DR, Hildebrandt T, Klimek P, Mitchell JE, Berg KC (2015). The eating disorder assessment for DSM-5 (EDA-5): development and validation of a structured interview for feeding and eating disorders. Int J Eating Disord.

[51] First MBS RL, Gibbon M, Williams JBW (2002). Structured clinical interview for DSM-IV-TR axis I disorders, research version, patient edition. (SCID-I/P).

[52] Company MLI (1959). New weight standards for men and women. Stat Bull.

[53] Fairburn CG, Beglin S (2008). Eating disorder examination questionnaire (EDE-Q 6.0). In: Fairburn CG, editor. Cognitive behavior therapy and eating disorders.

[54] Fairburn CG, Bohn K (2008). Clinical impairment assessment questionnaire (CIA 3.0). In: Fairburn CG, editor. Cognitive behavior therapy and eating disorders.

[55] Beck AT, Ward CH, Mendelson M, Mock J, Erbaugh J (1961). An inventory for measuring depression. Arch Gen Psychiatry.

[56] Beck AT, Steer RA (1993). Beck anxiety inventory manual.

[57] Spielberger CD, Gorsuch RL, Lushene R, Vagg PR, Jacobs GA (1983). Manual for the state-trait anxiety inventory.

[58] Cloninger CR (1994). The temperament and character inventory (TCI): a guide to its development and use.

[59] American Psychiatric Association. Diagnostic and Statistical Manual of Mental Disorders. 5th ed. Washington, DC. 2013.

[60] Bardone-Cone AM, Hunt RA, Watson HJ (2018). An overview of conceptualizations of eating disorder recovery, recent findings, and future directions. Curr Psychiatr Rep.

[61] Bardone-Cone AM, Harney MB, Maldonado CR, Lawson MA, Robinson DP, Smith R (2010). Defining recovery from an eating disorder: conceptualization, validation, and examination of psychosocial functioning and psychiatric comorbidity. Behav Res Ther.

[62] Mond JM, Hay PJ, Rodgers B, Owen C (2006). Eating disorder examination questionnaire (EDE-Q): norms for young adult women. Behav Res Ther.

[63] Steinhausen HC, Grigoroiu-Serbanescu M, Boyadjieva S, Neumärker KJ, Metzke CW (2009). The relevance of body weight in the medium-term to long-term course of adolescent anorexia nervosa. Findings from a multisite study. Int J Eating Disord.

[64] Lock J, Agras WS, Le Grange D, Couturier J, Safer D, Bryson SW (2013). Do end of treatment assessments predict outcome at follow-up in eating disorders?. Int J Eating Disord.

[65] Kästner D, Löwe B, Weigel A, Osen B, Voderholzer U, Gumz A (2018). Factors influencing the length of hospital stay of patients with anorexia nervosa - results of a prospective multi-center study. BMC Health Serv Res.

[66] Ratnasuriya RH, Eisler I, Szmukler GI, Russell GF (1991). Anorexia nervosa: outcome and prognostic factors after 20 years. Br J Psychiatr.

[67] Bulik CM, Sullivan PF, Fear JL, Joyce PR (1997). Eating disorders and antecedent anxiety disorders: a controlled study. Acta Psychiatr Scand.

[68] Kaye WH, Bulik CM, Thornton L, Barbarich N, Masters K (2004). Comorbidity of anxiety disorders with anorexia and bulimia nervosa. Am J Psychiatry.

[69] Marzola E, Porliod A, Panero M, De-Bacco C, Abbate-Daga G (2020). Affective temperaments and eating psychopathology in anorexia nervosa: which role for anxious and depressive traits?. J Affect Disord.

[70] Steinglass JE, Sysko R, Glasofer D, Albano AM, Simpson HB, Walsh BT (2011). Rationale for the application of exposure and response prevention to the treatment of anorexia nervosa. Int J Eat Disord.

[71] Cardi V, Leppanen J, Mataix-Cols D, Campbell IC, Treasure J (2019). A case series to investigate food-related fear learning and extinction using in vivo food exposure in anorexia nervosa: a clinical application of the inhibitory learning framework. Eur Eating Disord Rev.

[72] Steinglass JE, Glasofer DR, Dalack M, Attia E. Between wellness, relapse, and remission: Stages of illness in anorexia nervosa. Int J Eat Disord. 2020;53(7):1088–96. 10.1002/eat.23237 PMC7384605 32031292

[73] Berends T, Boonstra N, van Elburg A (2018). Relapse in anorexia nervosa: a systematic review and meta-analysis. Curr Opinion Psychiatr.

[74] Rigaud D, Pennacchio H, Bizeul C, Reveillard V, Verges B (2011). Outcome in AN adult patients: a 13-year follow-up in 484 patients. Diabetes Metab.

[75] Schebendach J, Mayer LE, Devlin MJ, Attia E, Walsh BT (2012). Dietary energy density and diet variety as risk factors for relapse in anorexia nervosa: a replication. Int J Eating Disord.

[76] Schebendach JE, Mayer LE, Devlin MJ, Attia E, Contento IR, Wolf RL (2008). Dietary energy density and diet variety as predictors of outcome in anorexia nervosa. Am J Clin Nutr.

