# Supplementary material for: Predictors of illness course and health maintenance following inpatient treatment among patients with anorexia nervosa
Source: J Eat Disord. 2020 Dec 2;8:69. doi: 10.1186/s40337-020-00348-7 (PMC7709230; doi:10.1186/s40337-020-00348-7)
Supplement: Supplementary file 1 — Additional file 1: Supplemental Table 1. Demographics and Clinical Characteristics of Participants and Non-Contributors [file 40337_2020_348_MOESM1_ESM.docx]

**Supplemental Table 1.** *Demographics and Clinical Characteristics of Participants and Non-Contributors.*

|  | **Participants**  **n = 168** | **Non-Contributors**  **n = 24** | **Test Statistic** |
| --- | --- | --- | --- |
| Age (years) | 26.02 ± 8.20 | 26.58 ± 7.65 | *t*(190) = -0.32*, p* = 0.75 |
| Sex, female, n (%) | 164 (98%) | 22 (92%) | *X*^2^ (1, *N* = 192) = 2.46*, p* = 0.12 |
| Race, Caucasian, n (%) | 148 (88%) | 20 (83%) | *X*^2^ (4, *N* = 192) = 2.72*, p* = 0.61 |
| Admission BMI (kg/m^2^) | 16.00 ± 1.86 | 15.36 ± 1.57 | *t*(190) = 1.61*, p* = 0.11 |
| Discharge BMI (kg/m^2^) | 20.02 ± 1.46 | 19.01 ± 1.77 | *t*(190) = 3.08, ***p* = 0.002** |
| Duration of illness (years) | 9.36 ± 7.70 | 10.32 ± 7.55 | *t*(188) = -0.57, *p =* 0.57 |
| Duration of hospitalization (days) | 78.82 ± 43.22 | 67.25 ± 34.27 | *t*(190) = 1.26, *p* = 0.21 |
| AN subtype, binge-purge, n (%) | 82 (49%) | 17 (71%) | *X*^2^ (1, *N* = 192) = 4.08*,* ***p* = 0.04** |

BMI = body mass index. Bold values = *p* < .05.
